# Neuropsychological and functional outcomes in recent-onset major depression, bipolar disorder and schizophrenia-spectrum disorders: a longitudinal cohort study

**DOI:** 10.1038/tp.2015.50

**Published:** 2015-04-28

**Authors:** R S C Lee, D F Hermens, S L Naismith, J Lagopoulos, A Jones, J Scott, K M Chitty, D White, R Robillard, E M Scott, I B Hickie

**Affiliations:** 1 Clinical Research Unit, Brain and Mind Research Institute, University of Sydney, Sydney, NSW, Australia; 2 Academic Psychiatry, Institute of Neuroscience, Newcastle University, Newcastle, UK; 3 Centre for Affective Disorders, Institute of Psychiatry, London, UK; 4 St Vincent's Hospital, University of Notre Dame, Sydney, NSW, Australia

## Abstract

Functional disability is the lead contributor to burden of mental illness. Cognitive deficits frequently limit functional recovery, although whether changes in cognition and disability are longitudinally associated in recent-onset individuals remains unclear. Using a prospective, cohort design, 311 patients were recruited and assessed at baseline. One hundred and sixty-seven patients met eligibility criteria (M=21.5 years old, s.d.=4.8) and returned for follow-up (M=20.6 months later, s.d.=7.8). Two-hundred and thirty participants were included in the final analysis, comprising clinically stable patients with major depression (*n*=71), bipolar disorder (BD; *n*=61), schizophrenia-spectrum disorders (*n*=35) and 63 healthy controls. Neuropsychological functioning and self-rated functional disability were examined using mixed-design, repeated-measures analysis, across diagnoses and cognitive clusters, covarying for relevant confounds. Clinical, neuropsychological and functional changes did not differ between diagnoses (all *P*>0.05). Three reliable neuropsychological subgroups emerged through cluster analysis, characterized by psychomotor slowing, improved sustained attention, and improved verbal memory. Controlling for diagnosis and changes in residual symptoms, clusters with improved neuropsychological functioning observed greater reductions in functional disability than the psychomotor slowing cluster, which instead demonstrated a worsening in disability (*P*<0.01). Improved sustained attention was independently associated with greater likelihood of follow-up employment (*P*<0.01). Diagnosis of BD uniquely predicted both follow-up employment and independent living. Neuropsychological course appears to be independently predictive of subjective and objective functional outcomes. Importantly, cognitive phenotypes may reflect distinct pathophysiologies shared across major psychiatric conditions, and be ideal targets for personalized early intervention.

## Introduction

Early characterization and treatment of cognitive deficits in major psychiatric disorders have the potential to prevent progression to more severe psychopathology^1, 2^ and functional disability.^2, 3, 4, 5, 6^ This notion is supported by data showing that those who go on to develop major depression (MD),^7, 8^ bipolar disorder (BD)^9^ and schizophrenia-spectrum disorder (SZ)^7, 8^ have poorer cognitive functioning in childhood than those who do not. Contrary to this body of evidence, data from other BD cohorts have identified superior cognitive functioning in those who later develop mania compared with those who remain healthy.^8, 10^ Nevertheless, the case for a premorbid or neurodevelopmental origin to cognitive dysfunction in psychiatric disorders is additionally supported by data showing that more pronounced neuropsychological dysfunction in established cases confers a greater risk of relapse in depression^11^ and psychosis.^12^ Despite widespread and persisting cognitive dysfunction in those with psychotic and mood disorders,^13, 14, 15^ and its effects on poorer prognosis and functional outcomes,^16, 17, 18^ the majority of studies have focused on well-established illness rather than recent-onset cases. Prioritizing research efforts toward recent-onset disorders is motivated by findings demonstrating that cognitive deficits in first-episode MD,^19^ BD^20^ and SZ^21^ contribute to functional disability independently of clinical and diagnostic considerations.^22^ The majority of published studies have relied on cross-sectional designs, whereas longitudinal studies have the unique ability to determine causative mechanisms, and can obviate between- and within-study heterogeneity frequently confounding cross-sectional studies.^20^

At a group level, it is now widely accepted that cognitive impairment in SZ remains relatively stable following the first episode of illness,^23^ at least until older adulthood.^24^ By contrast, there remains little consensus regarding the longer-term trajectory of cognitive functioning in mood disorders. The majority of longitudinal studies in MD have been very short term, with just two controlled studies conducted over a follow-up period of at least 1 year. One study found improved verbal memory,^25^ whereas another study identified psychomotor speed and executive functioning gains.^26^ Results in BD are also mixed, with studies finding improved^27, 28^ and stable cognitive deficits,^29, 30^ and other studies finding focal declines.^31, 32^ Thus, the neuropsychological trajectory in mood disorders appears heterogeneous, and the mechanisms of change remain unclear. Further, studies have been limited by using the Global Assessment of Functioning Scale, conflating functional outcome with symptomatology.^33^ Among the studies including other measures of functional outcome, none collected data at two time points. Importantly, only two longitudinal studies have examined recent-onset mood disorders in young adults.^26, 28^ Targeting early adulthood is critical given that interventions delivered during this period will likely have a greater impact on years lived with disability than focusing on later stages of the lifespan. Examining recent-onset cohorts also affords the added advantage of being able to circumvent the effects of prolonged medication use. On the whole, the above literature suggests that there is considerable heterogeneity in neuropsychological trajectories across MD and BD, and whether this differs from SZ, particularly in recent-onset cases, remains unknown. Furthermore, whether these trajectories can predict functional changes is unclear. Clarifying the time course and functional impact of cognitive deficits in mood disorders, compared with SZ, have implications for the understanding of the pathophysiology that may be shared across mood and psychotic disorders,^34, 35^ and for the development of novel and personalized early interventions.^36^ The identification of cognitive subgroups has already been successfully demonstrated in cross-sectional samples of MD,^37^ BD^38^ and SZ,^39^ and it remains to be empirically determined whether this can be extended to longitudinal samples that cut across traditional diagnostic boundaries. In addition, our understanding of whether the functional implications of neuropsychological dysfunction reflect real-world outcomes such as employment remains limited.

Here we sought to clarify the longitudinal course of neuropsychological functioning in clinically stable adolescents and young adults recently diagnosed with MD or BD, compared with a broadly defined SZ cohort at a similarly early stage of illness. We investigated whether neuropsychological changes and/or stability are shared across disorders, and potentially masked by traditional diagnostic classifications through a data-driven approach using cluster analysis. Finally, we examined whether neuropsychological changes are associated with changes in functional disability and, secondarily, with objective functional outcomes, such as employment.

## Materials and methods

### Participants

Participants were consecutively recruited from a specialized youth mental health clinic at the Brain and Mind Research Institute.^22, 40^ Inclusion criteria included persons aged 12–35 years presenting with a mood and/or psychotic syndrome. Healthy control (HC) participants were recruited from the same metropolitan region with no psychiatric or substance dependence history. HC subjects were recruited to serve as a comparison cohort from the same catchment area to control for socioeconomic characteristics that covary with geographic location. Participants were excluded if they had a neurological condition, current substance dependence (to eliminate the confound of acute substance use or withdrawal), insufficient English language skills or intellectual disability. The study was approved by the University of Sydney Human Research Ethics Committee, and all participants (or guardians, if participants were <16 years) gave written informed consent. Data included in the current study were collected between 9 July 2008 and 10 February 2014.

### Procedure

Psychiatrists and research psychologists conducted assessments at baseline and follow-up. Clinical diagnoses were determined by psychiatrists at both time points in accordance with Diagnostic and Statistical Manual of Mental Disorders, 4th edition, Text Revision. Follow-up clinical diagnoses were formally corroborated through case review by a board-registered psychologist according to the Structured Clinical Interview For DSM-IV-TR Axis I Disorders (non-patient edition).^41^ Any discrepancies were resolved by consensus between the treating psychiatrist and experienced research psychologists (see Supplementary Table 1 for diagnostic breakdown). We acknowledge that some may not consider psychotic disorder not otherwise specified to fall under ‘schizophrenia' as narrowly defined. However, we classified psychotic disorder not otherwise specified into a more broadly defined ‘schizophrenia-spectrum' cohort in the current study, as previously done in the literature.^42, 43^ All patients continued to receive ‘treatment as usual' between assessments with no interference to their prescribed course of treatment.

### Measures

Clinical symptoms were rated on the Brief Psychiatric Rating Scale,^44^ using empirically derived symptom (min–max) subscores,^45^ namely, depression (6–42), mania (7–49), positive symptoms (7–49), negative symptoms (5–35) and disorientation (2–14). Minimum scores were indicative of nil symptoms, whereas a single multiplication of the minimum score would correspond to an increase sequentially from none, very mild, mild, moderate, moderately severe, severe and finally to extremely severe (for example, depression of 18 would equate to ‘mild' severity).

Neuropsychological measures were chosen on the basis of sound validity and reliability,^46^ relevance to the diagnoses under study,^19, 20, 21^ overlap with the instruments used in the Measurement and Treatment Research to Improve Cognition in Schizophrenia initiative,^47, 48, 49^ and their degree of co-variation with one another (that is, we avoided highly correlated measures as this would artificially skew cluster solutions). Premorbid intellectual functioning (premorbid IQ) was estimated using the Wechsler Test of Adult Reading (WTAR)^50^ or Wide Range Achievement Test—fourth edition (WRAT-4; for participants <16 years).^51^ Psychomotor speed was measured using Trail Making Test—Part A (TMT-A).^52^ Verbal learning and memory were indexed using Logical Memory I and Logical Memory II Percent Retention (LM-I and LM-Ret).^53^ Sustained attention, visual learning and memory, and conceptual flexibility were assessed using Rapid Visual Processing Hits A′ (RVP-A′), Paired Associate Learning-adjusted errors (PAL) and Intra-/Extradimensional shift test-total errors (IED), respectively, from the Cambridge Neuropsychological Test Automated Battery (CANTAB).^54^ Verbal fluency was examined using the Controlled Oral Word Association Test (letters F, A and S; FAS).^55^ All neuropsychological raw scores were standardized into *z*-scores (higher scores denoted better performance) based on normative data^50, 53, 54, 56, 57, 58^ with established reliability and validity.^46^ This was to control for age-related changes in cognitive development, given the current age range coinciding with a critical period of cognitive and brain development.^46^

Self-ratings of disability and quality of life were obtained using the total scores from the World Health Organization Disability Assessment Scale version 2.0 (WHODAS-II)^59^ and World Health Organization Quality of Life (WHOQoL-BREF) Scale,^60^ respectively. A higher WHODAS-II score denoted greater functional disability, whereas a higher WHOQoL score denoted better quality of life. For the secondary analyses, objective real-world outcomes at follow-up were ascertained through interview and questionnaires probing information relating to employment and/or study, and relationship- and independent living statuses.

### Statistical analyses

Statistical analyses were conducted using SPSS Version 20 (SPSS, Chicago, IL, USA). One-way analyses of variance and *χ*^2^-tests were conducted to examine baseline differences. Mixed-design, repeated-measures analyses of covariance were conducted to assess overall change and diagnosis-by-time interactions in clinical, neuropsychological and functional characteristics, while controlling for age and gender.

Monte Carlo simulation has previously shown that the best-performing clustering algorithm was a two-step process involving (a) determining membership through hierarchical cluster analysis and (b) assigning group membership through *k*-means clustering.^61^ Therefore, a hierarchical cluster analysis was conducted on neuropsychological change scores to identify distinct and internally homogeneous change subgroups using Ward's method of minimum variance with squared Euclidean distance.^37, 62^ An optimal cluster solution was determined from the agglomeration schedule and dendrogram, and *k*-means clustering was conducted to segregate patients into clusters. A good cluster solution is one where the data separates into reliable (that is, stable) and externally valid (that is, meaningful) groups.^63^ Accordingly, we examined the stability of clusters through multiple methods as previously recommended^64^ by conducting further *k*-means clustering on data after (a) case order was randomized, and (b) a random 50% subsample was generated. Cluster membership was deemed stable when a high percentage of cases were reassigned to the same clusters.

Baseline demographic and clinical characteristics between clusters were examined using analyses of variance and *χ*^2^-tests. Mixed-design repeated-measures analyses of covariance were used to examine overall changes and cluster-by-time interactions in clinical symptoms, covarying for age, gender and diagnosis. This analysis was repeated for neuropsychological functioning, functional disability and quality of life, while additionally covarying for differential changes in symptoms between clusters. Secondarily, we examined whether the functional relevance of neuropsychological trajectories would extend to objective real-world outcomes through logistic regression, controlling for the same covariates. Specifically, we examined whether clusters would be predictive of (1) employment in those who were not in full-time education, (2) relationship status and (3) independent living in adults (that is, having moved out of the family home). We included only those who were not in full-time education in our first analysis, as we could not reasonably define participants who were in full-time education, but not employed, as occupationally disengaged in the same way as individuals who were both not in full-time education and not employed. Further, we hypothesized that living independently away from the family home would likely rely on an independent source of remuneration. As such, we added employment as a covariate of independent living status. Significance was set at 0.05 (two-sided). All contrasts were Bonferroni-adjusted to control for type I error rate.

## Results

### Baseline characteristics by diagnosis

At baseline, 311 patients were recruited and assessed, with 197 participants successfully returning for follow-up (Supplementary Figure 1). Those who were lost to follow-up did not differ on age, sex, educational attainment, symptomatology, functional disability or quality of life, although they had lower premorbid IQ (100.65 vs 104.45, F=9.31, *P*<0.01), poorer verbal learning (LM-I, F=6.93, *P*<0.01) and worse conceptual flexibility (IED, F=4.84, *P*<0.05). No other neuropsychological variables differed between those who returned and those who did not.

Of those who returned for follow-up, 30 patients were deemed ineligible and were excluded from the final analysis (Supplementary Figure 1 for reasons of exclusion). This left a sample of 167 patients who were case-reviewed and consensus-rated for a formal diagnosis. The final sample of 167 patients comprised 71 MD, 61 BD and 35 SZ cases. An HC sample was also recruited and assessed at baseline (*n*=63). All patients were reassessed, on average, 20.6 months later (s.d.=7.8, range=6–48 months). Most participants were reassessed between 12 and 36 months after the baseline assessment (*n*=157), with only 3% (*n*=5) falling below and above this threshold. Importantly, there were no significant differences in follow-up period between diagnoses.

Mean age of participants differed between groups (F=12.8, *P*<0.001; Table 1), with MD participants being younger than BD (*P*<0.01), SZ (*P*<0.05) and HC cases (*P*<0.001). Distribution of sex differed between groups (*χ*^2^=17.7, *P*<0.001), with SZ having a higher proportion of males than the other three groups (all *P*<0.05). Average years of education differed between groups (F=13.0, *P*<0.001), with HC participants being more educated than the other three groups (all *P*<0.01). Average premorbid IQ (s.d.) was 104.6 (9.4), with no significant differences between groups.

Age of clinical presentation differed significantly between diagnostic groups (F=7.9, *P*<0.01), with MD participants presenting at an earlier age than BD and SZ groups (all *P*<0.01). The length of time between presentation and baseline assessment did not differ between diagnostic groups. Qualitatively, mean symptom scores all fell in the very mild-to-mild range across diagnoses, corroborating the clinically stable status of our cohort. Groups differed in residual positive symptoms at baseline (F=7.9, *P*<0.05), with SZ having more severe symptoms than MD (*P*<0.05) and BD (*P*<0.01). This is consistent with differences in history of psychotic features between groups (*χ*^2^=82.0, *P*<0.001), with a higher proportion of psychotic presentations in the SZ group than the MD or BD groups (all *P*<0.05). Antipsychotic use (*χ*^2^=26.2, *P*<0.001) and mood stabilizer use (lithium/anticonvulsant; *χ*^2^=23.4, *P*<0.001) were different between groups, with these being more common in SZ and BD groups (all *P*<0.05), respectively.

**Figure 1:**
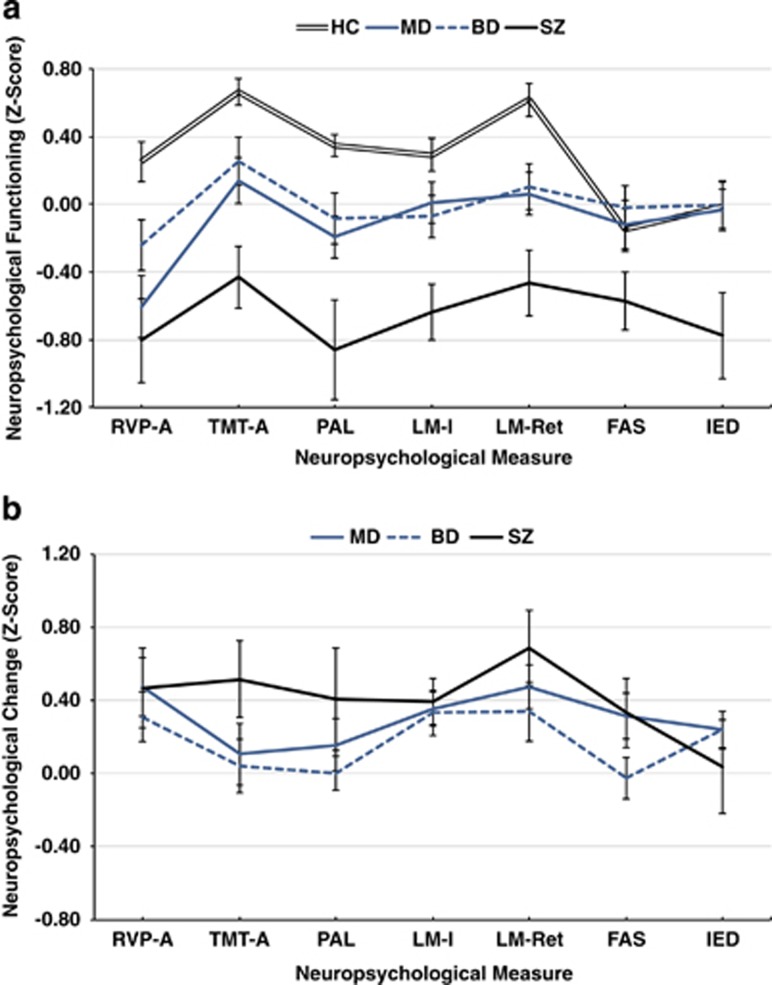
Mean standardized neuropsychological performance (±s.e.) at baseline (**a**) and mean standardized neuropsychological change (±s.e.) at follow-up (**b**) by diagnosis. At baseline (**a**), neuropsychological functioning was significantly different between diagnoses across all measures (except FAS). Specifically, patients with schizophrenia-spectrum disorder (SZ) performed worse than healthy controls (HCs; *P*<0.05) on all measures (except FAS). Major depression (MD) and bipolar disorder (BD) each performed worse than HC (*P*<0.05) on RVP-A, PAL and LM-Ret. Separately, MD and BD performed worse than HC (*P*<0.05) on TMT-A and LM-I, respectively. MD and BD each outperformed SZ on TMT-A, PAL, LM-I, LM-Ret and IED (*P*<0.05). MD and BD did not differ from each other on any measure (*P*>0.05). At follow-up (**b**), LM-Ret was the only measure improving significantly over time across diagnoses (*P*<0.05). All group × time interactions were nonsignificant (*P*>0.05). FAS, Controlled Oral Word Association Test; IED, Intra-/Extradimensional shift test-total errors; LM-I, Logical Memory I; LM-Ret, Logical Memory II Percent Retention; PAL, Paired Associates Learning-adjusted errors; RVP-A, Rapid Visual Processing Hits A′ TMT-A′, Trail Making Test—Part A.

Cognitively, diagnostic groups differed on all measures at baseline except FAS (all *P*<0.05; Figure 1 and Supplementary Table 2). The SZ participants were most impaired, performing worse than HC on all measures except FAS (*P*<0.05), whereas MD and BD participants performed at an intermediate level between HC and SZ.

There were no baseline differences in self-rated functional disability (WHODAS-II) or quality of life (WHOQoL) between diagnostic groups (all *P*>0.05).

### Clinical, neuropsychological and functional changes by diagnosis

**Figure 2:**
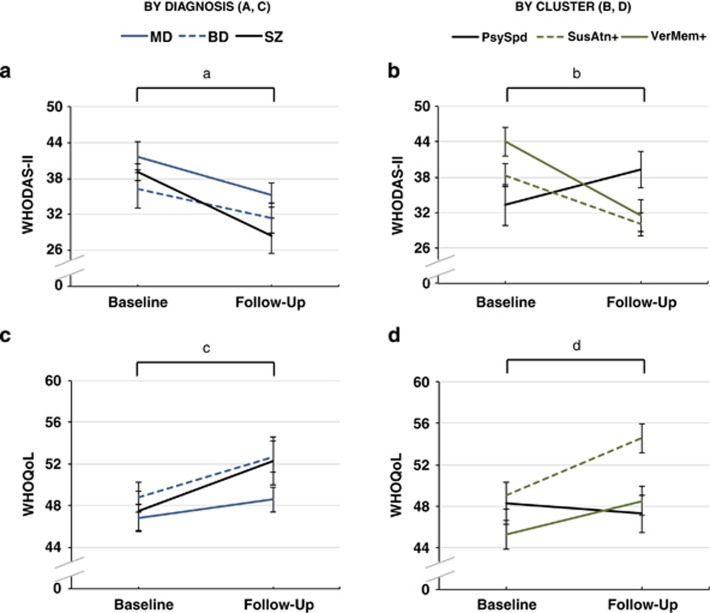
Mean baseline and follow-up WHODAS-II (**a**, **b**) and WHOQoL (**c, d**) scores (±s.e.). ^a^Overall change and group × time interaction is nonsignificant (*P*>0.05). ^b^Overall change is nonsignificant (*P*>0.05), whereas group × time interaction is significant (*P*<0.01). Pairwise comparisons showed that the SusAtn+ and VerMem+ clusters were each changing at significantly different rates from the PsySpd− cluster (*P*<0.01 and *P*<0.001, respectively). No other pairwise comparison was significant. ^c^Overall change and group × time interaction is nonsignificant (*P*>0.05). ^d^Overall change and group × time interaction is nonsignificant (*P*>0.05). WHODAS-II, World Health Organization Disability Assessment Schedule Version 2.0; WHOQoL, World Health Organization Quality of Life (BREF) Scale.

There were no overall changes or diagnosis-by-time interactions in symptoms. Cognitively, overall improvements were only found for LM-Ret (F=4.9, *P*<0.05). To assess for the influence of practice effects, follow-up interval was correlated with LM-Ret change, as more pronounced improvements would theoretically be associated with shorter follow-up intervals if practice effects were at play. However, this was nonsignificant (*r*=−0.062, *P*=0.434) and argues against the presence of practice effects. All diagnosis-by-time interactions for cognitive functioning were nonsignificant. Functionally, there were no significant overall changes in WHODAS-II, WHOQoL or diagnosis-by-time interactions (Figure 2).

### Cluster analysis of neuropsychological change

Hierarchical clustering identified three distinct clusters (Supplementary Figure 2 and Supplementary Table 2), showing predominant reductions in psychomotor speed (PsySpd−, *n*=36), improvement in sustained attention (SusAtn+, *n*=70) and improvement in verbal memory (VerMem+, *n*=56). Two randomized data sets generated largely similar clusters with 93.8 and 87.7% of all cases being assigned to the same clusters. Moreover, a randomly generated subsample of half of participants produced similar clusters, with 80.0% of participants being assigned to the same clusters and the most pronounced changes found on the same neuropsychological measures (TMT-A, RVP-A and LM-Ret).

### Demographic and clinical characteristics of clusters

At baseline (Table 2), clusters did not significantly differ on age, sex, premorbid IQ, educational attainment, clinical symptoms, medication use or length of time between presentation and baseline assessment. Notably, follow-up interval or diagnosis did not differ between clusters (Supplementary Table 3).

There were no overall changes in symptoms. However, when examining differential changes at follow-up (Supplementary Figure 3), discrepancies between clusters emerged for both positive (F=4.2, *P*<0.05) and negative symptoms (F=4.1, *P*<0.05). Specifically, the VerMem+ cluster showed improvements in positive and negative symptoms, whereas the PsySpd− cluster showed worsening of negative symptoms, while their positive symptoms remained stable. The differential changes in symptoms between these two clusters were statistically significant (*P*<0.01). The rate of change in the SusAtn+ cluster for both positive and negative symptoms did not differ from any of the other two clusters.

### Functional disability by clusters

Baseline functional disability differed between clusters (F=4.0, *P*<0.05), with the PsySpd− cluster reporting a lower level of disability at baseline compared with the VerMem+ cluster (*P*<0.05). Given clusters did not differ on any demographic or clinical factors, we conducted additional *post hoc* analyses on baseline neuropsychological scores to clarify the nature of the baseline difference in functional disability. Accordingly, the PsySpd− cluster was more impaired than the VerMem+ cluster on all three learning and memory measures (PAL, LM-I and LM-Ret, *P*<0.01). No other cognitive functions differed between these two clusters.

At follow-up, WHODAS-II change differed between clusters (F=5.6, *P*<0.01; Figure 2). Specifically, SusAtn+ and VerMem+ clusters were each changing at different rates compared with the PsySpd− cluster (*P*<0.01), such that improved sustained attention and improved verbal memory were associated with reduced functional disability, whereas psychomotor slowing was linked to a worsening of functional disability. Clinical symptom changes did not significantly predict WHODAS-II change over and above cluster membership (*P*>0.05). By comparison, WHOQoL changes did not differ between clusters (*P*>0.05).

### Secondary analyses of real-world outcomes at follow-up

Of those not currently in full-time education, those in the SusAtn+ cluster were more likely to be employed at follow-up than those in the PsySpd− cluster (odds ratio (OR)=7.3, 95% confidence interval (CI), 2.0–26.8, *P*<0.005; Table 3), with improved sustained attention contributing an additional 9.2% to the variance in follow-up employment (Δ*R*^2^=0.092, *P*<0.01). Cluster membership did not independently predict the likelihood of being in a relationship or living independently from family members (*P*>0.05). By comparison, being female was a significant predictor of being in a relationship at follow-up (OR=3.12, 95% CI, 1.4–6.9, *P*<0.01). Of note, although diagnosis did not differentiate between changes in functional disability or quality of life, being diagnosed with BD increased the likelihood of follow-up employment (OR=5.37, 95% CI, 1.6–18.5, *P*<0.01) and independent living (OR=3.39, 95% CI, 1.0–11.1, *P*<0.05) than being diagnosed with SZ.

## Discussion

At a group level, the early course of neuropsychological functioning in MD and BD is generally comparable to a broadly defined cohort of SZ, whereby functioning remains largely stable. This stability is generally consistent with findings within the first-episode psychosis literature.^65^ Verbal memory was the only current exception, which improved at follow-up across diagnoses, and corroborates data in recent-onset MD^26^ and BD.^28^ Interestingly, prior investigations examining the association between clinical and neuropsychological change in MD have consistently found that changes in depression are correlated with changes in verbal memory,^66^ in keeping with meta-analytic evidence supporting the same association in cross-sectional studies of first-episode MD.^19^ Given that residual depressive symptoms did not significantly change in the current study, any potential relationship between reductions in depression and improvement in verbal memory may have been obscured by a restriction in range and warrants further examination in future studies.

An alternative approach to interrogate the longitudinal course of neuropsychological functioning was to use a data-driven cluster analysis to help identify potential discrete cognitive pathways obscured by simple diagnostic aggregation.^67^ Three distinct and reliable neuropsychological subgroups emerged, representing predominantly psychomotor slowing and improvements in sustained attention and verbal memory. Diagnostic makeup did not differ between clusters, underscoring the notion that the course of neuropsychological functioning does not appear to be diagnosis specific. Further analyses revealed that changes in positive and negative symptoms differed between clusters, with improved verbal memory associated with reductions in residual positive and negative symptoms, whereas psychomotor slowing was linked to a worsening of negative symptoms. This finding converges with meta-analytic data in first-episode psychosis, showing an influence of both positive and negative symptom changes on the severity of cognitive dysfunction.^65^

Importantly, the external validity of the neuropsychological clusters was further supported by their associations with functional changes. Specifically, improved verbal memory and sustained attention were each coupled with greater reductions in self-rated functional disability than psychomotor slowing, over and above the effects of diagnosis and symptom alleviation. Surprisingly, another study investigating the association between cognitive and functional changes also found that improved verbal memory and sustained attention were correlated with reduced functional impairments, albeit in older cases with longstanding BD.^27^ The current findings show that this relationship also holds for the full spectrum of major mood and psychotic illnesses, even at the early stages of illness. Moreover, it highlights the incremental value of neuropsychology over and above diagnostic considerations in predicting the course of functional changes. It is interesting to note that the psychomotor slowing cluster presented with less functional disability than the improved verbal memory cluster at baseline. Our *post hoc* analyses revealed that this difference appeared to be related to learning and memory being more superior in the psychomotor slowing cluster and converges with evidence showing that episodic memory seems to be most strongly linked to psychosocial functioning compared with other neuropsychological functions.^6^

Contrary to functional disability, changes in quality of life in recent-onset MD, BD and SZ do not appear to be correlated with neuropsychological changes. Instead, the clusters with improved quality of life were associated with reductions in positive and negative symptomatology, consistent with the first-episode psychosis literature showing that quality of life appears to be more strongly related to psychopathology than neuropsychological functions.^68^ This distinction highlights the importance of examining clinical phenotypes separately from diagnostic entities, as certain symptom dimensions are evidently critical to functional outcomes, such as quality of life, irrespective of diagnosis.

Secondarily, individuals who were not in full-time education, but whose sustained attention and self-rated functional disability improved, were more likely to be employed at follow-up than those whose experienced psychomotor slowing and functional decline. Few previous longitudinal studies had specifically examined real-world functional outcomes,^22^ and this finding lends preliminary support to the ecological validity of self-rated functional disability in the domain of occupational functioning. By comparison, a diagnosis of BD also increased the likelihood of follow-up employment, as well as the odds of living independently from family members, compared with having a diagnosis of a SZ. Accordingly, despite diagnosis contributing negligibly to the course of self-rated functional disability, it appears to have specific utility in predicting real-world role, as well as independent living, outcomes. This is consistent with a burgeoning view within the literature that there appears to be a subset of BD cases with undetectable premorbid decline in cognitive functioning.^69, 70^ Instead, at least in the early stages of illness, BD cases may present with psychosocial functions superior to healthy comparison subjects, which may have accounted for the more favorable employment and independent living outcomes of BD cases in the current sample.^38, 71^

Conceptually, the current finding that diagnostic classification as broadly defined is unable to discriminate the longitudinal course of clinical symptoms, neuropsychological functioning or self-rated functional disability has important implications for psychiatric nosology. It contributes to an emerging view within psychiatry of a need to refocus efforts toward less arbitrarily defined and more biologically valid phenotypes under the Research Domain Criteria framework.^72^ Solely diagnostic approaches to study designs continue to obstruct efforts to clarify the underlying structure of mental illness, as these involve classifying individuals on various potentially unrelated illness characteristics likely to have separate underlying pathophysiologies.^73^ By comparison, ‘dimensional psychiatry' using cognitive phenotypes, as exercised in the current study, is better equipped to isolate independent and internally consistent subgroups across diagnostic boundaries to better elucidate how these contribute to disease characteristics and progression.^67, 74^

The current study was limited by the absence of follow-up assessments for HCs. As such, we could not definitively rule out the effects of practice on repeated cognitive testing, although we did attempt to address this through *post hoc* analyses showing that potential cognitive changes secondary to practice were not supported by the data. Further, the length of follow-up in the present study was longer than the period conventionally expected to give rise to sizeable practice effects.^46^ Despite the length of follow-up being longer than previously conducted in MD studies, however, this duration was still shorter than most studies in BD, and greater lengths of follow-up would be needed to determine the longer-term changes associated with relapse and disease progression. The range of follow-up periods in the current study was also large, and should be more tightly restricted in future studies, although its effects on the current findings are likely to be small, if at all present, given that the length of follow-up was comparable across all diagnoses and clusters. Further, conducting neuropsychological follow-up over at least three time points would be necessary to establish the long-term trajectory of neuropsychological change or stability. Future studies may also consider examining whether certain neuropsychological tests are better at characterizing particular cognitive subgroups, whether multiple significant cognitive impairments would be associated with worse functional impairments, and whether more severe symptoms are associated with more multiple cognitive deficits. In addition, although neuropsychological clusters did not differ on medication usage, future studies would also benefit from more treatment-homogeneous samples to rule out the potential effects of psychotropic medications, or other treatment parameters, on the longitudinal trajectory of neuropsychological functioning. Finally, the loss of participants to follow-up was quite substantial in the current study, and it would be important to replicate the present findings in future, larger, multisite cohorts.

To our knowledge, this is the first prospective, longitudinal study to examine neuropsychological functioning across all three major psychiatric disorders, and is also one of the most statistically powered. In the medium term, neuropsychological functioning in recent-onset mood disorders does not appear to differ from that of schizophrenia-spectrum psychoses. Importantly, the current data suggest that interrogations based on traditional diagnostic groups are unlikely to yield insights into the cognitive architecture of major mood and psychotic disorders, as neuropsychological changes are shared across, and obscured by, diagnostic boundaries. By comparison, defining cases according to cognitive factors appear to yield more internally consistent subgroups. Neuropsychological changes appear to be related to residual changes in positive and negative symptoms, suggesting that therapies targeting these specific clinical phenotypes may be useful in preventing or slowing down cognitive deterioration in a subset of patients with major mood or psychotic illness. Importantly, cognitive subgroups are functionally relevant and distinct from clinical state, whereby improvements in cognitive functioning are independently associated with reductions in self-rated functional disability. Therefore, cognitive factors may be ideal targets for novel and personalized early interventions. Further, neuropsychological clusters have real-world relevance in terms of employment outcomes. Using data-driven approaches to examine shared cognitive phenotypes, thus, have the potential to reveal new insights into the pathophysiological changes that are common across recent-onset mood and schizophrenia-spectrum illnesses, and warrants more detailed multimodal genetic, metabolic and neurobiological investigations.

## Figures and Tables

**Table 1:** Sample characteristics at baseline by diagnosis

*Measures*	*MD (*n*=71)*		*BD (*n*=61)*		*SZ (*n*=35)*		*HC (*n*=63)*		*Inferential statistics*		
	*Mean*	*s.d.*	*Mean*	*s.d.*	*Mean*	*s.d.*	*Mean*	*s.d.*	*F*	P*-value*	*Contrasts*
*Demographic*											
Age	19.9	4.2	22.8	5.1	22.7	4.5	24.1	3.7	12.8^a^	<0.00	MD<BD, SZ, HC^b^
Education (years)	12.1	2.4	12.9	2.6	12.5	2.6	14.5	2.0	13.0	<0.00	MD, BD, SZ<HC^b^
Premorbid IQ	104.8	10.8	104.6	8.8	103.5	10.6	105.0	7.7	0.2	NS	
											
*Clinical*											
Age at presentation	19.0	3.9	21.7	4.8	21.9	4.6			7.9	<0.00	MD<BD, SZ^b^
BPRS depression	15.1	5.0	13.5	5.2	13.1	6.1			2.1	NS	
BPRS mania	9.8	4.0	10.6	4.4	10.3	4.9			0.6	NS	
BPRS positive	10.8	3.3	10.4	3.4	13.1	5.3			3.7^a^	0.03	MD, BD<SZ^b^
BPRS negative	9.8	4.0	10.6	4.4	10.3	4.9			3.1^a^	NS	
BPRS disorientation	2.2	0.5	2.2	0.5	2.6	1.1			2.0^a^	NS	
											
*Functional*											
WHODAS-II total	41.6	14.5	36.2	18.9	39.5	16.5			1.5	NS	
WHOQoL total	46.8	10.7	48.8	10.9	47.5	10.4			0.5	NS	
Follow-up interval (months)	20.7	7.7	19.9	7.6	21.8	8.2			0.7	NS	
	N	*%*	N	*%*	N	*%*	N	*%*	χ*^2^*	P*-value*	
*Demographic*											
Gender (female)	45	63.4	41	67.2	9	25.7	35	55.6	17.7	<0.00	MD, BD, HC>SZ^b^
											
*Clinical*											
Psychotic features	12	16.9	16	26.2	35	100.0			82.0^c^	<0.00	MD, BD<SZ^b^
											
*Medications*											
Any psychotropic	44	62.0	51	83.6	31	88.6			11.9	<0.00	MD<BD, SZ^b^
Antidepressant	37	52.1	30	49.2	12	34.3			3.1	NS	
Antipsychotic	19	26.8	34	55.7	27	77.1			26.2	<0.00	MD, BD<SZ^b^
Lithium or anticonvulsant	4	5.6	24	39.3	6	17.1			23.4^c^	<0.00	MD, SZ<BD^b^

Abbreviations: BD, bipolar disorder; BPRS, Brief Psychiatric Rating Scale; HC, healthy control; MD, major depression; NS, nonsignificant; SZ, schizophrenia-spectrum disorder; WHODAS-II, World Health Organization Disability Assessment Schedule Version 2.0; WHOQoL, World Health Organization Quality of Life (BREF) Scale.

a Welch's statistic correction for violation of homoscedasticity.

b Bonferroni-adjusted contrasts between individual diagnostic groups statistically significant (*P*<0.05).

c Fisher's exact test correction for cells with *n*<5.

**Table 2:** Sample characteristics at baseline by cluster

*Measures*	*PsySpd− (*n*=36)*		*SusAtn+ (*n*=70)*		*VerMem+ (*n*=56)*		*Inferential statistics*		
	*Mean*	*s.d.*	*Mean*	*s.d.*	*Mean*	*s.d.*	*F*	P*-value*	*Contrasts*
*Demographic*									
Age	22.1	6.0	21.6	4.5	20.8	4.4	0.9^a^	NS	
Education (years)	12.4	2.8	12.7	2.3	12.0	2.5	1.0	NS	
Premorbid IQ	106.6	10.4	104.0	8.4	103.3	11.3	1.3	NS	
									
*Clinical*									
Age at presentation	20.6	4.8	21.0	4.6	19.9	4.4	0.8	NS	
BPRS depression	13.8	5.5	13.9	5.4	14.6	5.2	0.4	NS	
BPRS mania	10.1	4.3	10.6	5.0	9.9	3.5	0.4	NS	
BPRS positive	10.7	3.6	11.0	3.8	11.8	4.4	0.9	NS	
BPRS negative	7.1	2.4	7.3	2.9	7.7	3.2	0.5	NS	
BPRS disorientation	2.1	0.5	2.3	0.7	2.3	0.9	0.7	NS	
									
*Functional*									
WHODAS-II total	33.2	18.6	38.3	15.0	44.0	16.9	4.0	0.02	PsySpd− <VerMem+^b^
WHOQoL total	48.3	11.9	49.0	10.3	45.3	10.1	1.9	NS	
Follow-up interval (months)	20.3	5.6	20.8	7.5	20.8	9.1	0.1	NS	
	N	*%*	N	*%*	N	*%*	χ*^2^*	P*-value*	
*Demographic*									
Gender (female)	23	63.9	41	58.6	30	53.6	1.0	NS	
									
*Clinical*									
Psychotic features	14	38.9	23	32.9	23	41.1	1.0	NS	
									
*Medications*									
Any psychotropic	27	75.0	52	74.3	42	75.0	0.0	NS	
Antidepressant	18	44.4	32	45.7	28	50.0	0.3	NS	
Antipsychotic	21	58.3	28	40.0	28	50.0	3.4	NS	
Lithium or anticonvulsant	8	22.2	11	15.7	13	23.2	1.3	NS	

Abbreviations: BPRS, Brief Psychiatric Rating Scale; NS, nonsignificant; PsySpd−, psychomotor speed decline; SusAtn+, sustained attention improvement; VerMem+, verbal memory improvement; WHODAS-II, World Health Organization Disability Assessment Schedule Version 2.0; WHOQoL, World Health Organization Quality of Life (BREF) Scale.

a Welch's statistic correction for violation of homoscedasticity.

b Bonferroni-adjusted contrasts between individual clusters statistically significant (*P*<0.05).

**Table 3:** Binary logistic regression models predicting follow-up employment, relationship status and independent living

*Employment*	Step 1* (Cox–*Snell,* R^*2*^*=0.13)*				Step 2** (Cox–*Snell,* R^*2*^*=0.22;* R^*2*^ change**)			
	*B (SE)*	*OR*	*95% CI*	P*-value*	*B (SE)*	*OR*	*95% CI*	P-*value*
Age	0.01 (0.05)	1.01	0.92–1.11	NS	0.04 (0.05)	1.04	0.93–1.16	NS
Sex (female)	0.22 (0.47)	1.24	0.49–3.14	NS	0.38 (0.53)	1.46	0.52–4.12	NS
								
*Diagnosis*								
MD	0.35 (0.62)	1.42	0.42–4.83	NS	0.68 (0.65)	1.97	0.55–7.11	NS
BD	1.68 (0.63)	5.37	1.55–18.54	0.008	2.04 (0.68)	7.67	2.03–28.93	0.003
Positive symptom change	−0.04 (0.06)	0.96	0.86–1.07	NS	−0.01 (0.06)	0.99	0.88–1.11	NS
Negative symptom change	0.02 (0.07)	1.02	0.88–1.17	NS	0.06 (0.08)	1.07	0.91–1.25	NS
								
*Cluster*								
SusAtn+					1.99 (0.66)	7.34	2.01–26.83	0.003
VerMem+					1.08 (0.71)	2.94	0.73–11.80	NS

*Relationship status*	Step 1** (Cox–*Snell,* R^*2*^*=0.12)*				Step 2** (Cox–*Snell,* R^*2*^*=0.13; R*^*2*^ *change*^*†*^)			
	*B (SE)*	*OR*	*95% CI*	P-*value*	*B (SE)*	*OR*	*95% CI*	P-*value*
Age	0.08 (0.04)	1.08	1.00–1.17	NS	0.08 (0.04)	1.09	1.00–1.18	0.041
Sex (female)	1.14 (0.40)	3.12	1.41–6.87	0.005	1.18 (0.41)	3.27	1.47 -7.28	0.004
								
*Diagnosis*								
MD	0.53 (0.57)	1.70	0.55–5.20	NS	0.61 (0.58)	1.84	0.60–5.68	NS
BD	0.90 (0.56)	2.46	0.82–7.40	NS	0.95 (0.57)	2.57	0.85–7.80	NS
Positive symptom change	−0.02 (0.05)	0.98	0.89–1.08	NS	−0.01 (0.05)	0.99	0.90–1.09	NS
Negative symptom change	0.10 (0.07)	1.11	0.97–1.26	NS	0.12 (0.07)	1.12	0.98–1.29	NS
								
*Cluster*								
SusAtn+					0.63 (0.49)	1.88	0.72–4.89	NS
VerMem+					0.52 (0.53)	1.67	0.59–4.73	NS

*Independent living*	Step 1*** (Cox–*Snell,* R^*2*^*=0.20)*				Step 2** (Cox–*Snell,* R^*2*^*=0.21; R*^*2*^ *change*^*†*^)			
	*B (SE)*	*OR*	*95% CI*	P*-value*	*B (SE)*	*OR*	*95% CI*	P-*value*
Age	0.19 (0.06)	1.21	1.09–1.35	0.000	0.19 (0.06)	1.21	1.08–1.35	0.001
Sex (female)	0.06 (0.43)	0.88	0.46–2.48	NS	0.04 (0.44)	1.04	0.44–2.44	NS
								
*Diagnosis*								
MD	0.95 (0.61)	2.59	0.79–8.54	NS	0.94 (0.62)	2.56	0.77–8.57	NS
BD	1.22 (0.60)	3.39	1.04–11.05	0.043	1.20 (0.61)	3.33	1.01–10.95	0.047
Positive symptom change	−0.07 (0.06)	0.94	0.84–1.05	NS	−0.08 (0.06)	0.93	0.83–1.04	NS
Negative symptom change	−0.02 (0.08)	0.98	0.84–1.15	NS	−0.03 (0.08)	0.97	0.83–1.14	NS
Employment	0.87 (0.42)	2.38	1.05–5.41	0.038	0.91 (0.43)	2.49	1.07–5.79	0.034
								
*Cluster*								
SusAtn+					−0.14 (0.57)	0.87	0.28–2.65	NS
VerMem+					−0.46 (0.61)	0.63	0.19–2.08	NS

Abbreviations: BD, bipolar disorder; MD, major depression; NS, nonsignificant; SusAtn+, sustained attention improvement; VerMem+, verbal memory improvement.

SZ was the reference group for diagnosis, and PsySpd− was the reference group for cluster.

*P<0.05, **P<0.01, ***P<0.001, ^†^Nonsignificant.

## Notes

IBH has led projects for health professionals and the community supported by governmental, community agency and drug industry partners (Wyeth, Eli Lily, Servier, Pfizer and AstraZeneca) for the identification and management of depression and anxiety. He has served on advisory boards convened by the drug industry in relation to specific antidepressants, including nefazodone, duloxetine and desvenlafaxine, and has participated in a multicenter clinical trial of agomelatine effects on sleep architecture in depression. He has participated in Servier-sponsored educational programs related to circadian-based therapies. DFH has previously received honoraria for educational seminars from Janssen-Cilag. The research by JS has been supported by funds provided to the Northumberland, Tyne and Wear National Health Service Trust by AstraZeneca and Janssen-Cilag for investigator-initiated studies on medication adherence. She has received honoraria for educational seminars supported by the pharmaceutical industry and has served on advisory boards convened by the pharmaceutical industry (including AstraZeneca, Janssen-Cilag, Lundbeck, Pfizer, Sanofi-Aventis and Servier). EMS has received honoraria for educational seminars related to the clinical management of depressive disorders supported by Servier and Eli-Lilly pharmaceuticals. She has also participated in a national advisory board for the antidepressant compound Pristiq, manufactured by Pfizer. The remaining authors declare no conflict of interest.
