# Supplementary material for: Neuropsychological and functional outcomes in recent-onset major depression, bipolar disorder and schizophrenia-spectrum disorders: a longitudinal cohort study
Source: Transl Psychiatry. 2015 Apr 28;5(4):e555–. doi: 10.1038/tp.2015.50 (PMC4462613; doi:10.1038/tp.2015.50)
Supplement: Supplementary Table 1 [file tp201550x1.docx]

**SUPPLEMENTARY TABLE 1.** Diagnostic breakdown according to DSM-IV-TR.

| Diagnosis | n / N |
| --- | --- |
| Major depressive disorder | 58 |
| Major depressive disorder with psychotic features | 13 |
| MD n | 71 |
| Bipolar I disorder | 11 |
| Bipolar I disorder with psychotic features | 12 |
| Bipolar II disorder | 24 |
| Bipolar II disorder with psychotic features | 4 |
| Bipolar disorder not otherwise specified | 10 |
| BD n | 61 |
| Schizophrenia | 16 |
| Schizoaffective disorder - depressive type | 3 |
| Schizoaffective disorder - bipolar type | 9 |
| Psychotic disorder not otherwise specified | 7 |
| SZ n | 35 |
| Total N | 167 |
