# Supplementary material for: Neuropsychological and functional outcomes in recent-onset major depression, bipolar disorder and schizophrenia-spectrum disorders: a longitudinal cohort study
Source: Transl Psychiatry. 2015 Apr 28;5(4):e555–. doi: 10.1038/tp.2015.50 (PMC4462613; doi:10.1038/tp.2015.50)
Supplement: Supplementary Table 2 [file tp201550x2.docx]

**SUPPLEMENTARY TABLE 2.** Standardised neuropsychological performance (z-scores ± SD).

| Measure | MD (n = 71) | | BD (n = 61) | | SZ (n = 35) | |
| --- | --- | --- | --- | --- | --- | --- |
|  | BL | FU | BL | FU | BL | FU |
| RVP-A | -0.60 ± 1.5 | -0.09 ± 1.3 | -0.24 ± 1.1 | 0.12 ± 1.2 | -0.81 ± 1.4 | -0.48 ± 1.0 |
| TMT-A | 0.13 ± 1.2 | 0.24 ± 1.2 | 0.26 ± 1.1 | 0.27 ± 1.2 | -0.46 ± 1.1 | 0.06 ± 0.9 |
| PAL | -0.14 ± 1.0 | 0.00 ± 0.9 | -0.02 ± 1.1 | -0.07 ± 1.2 | -0.93 ± 1.7 | -0.59 ± 1.4 |
| LM-I | 0.00 ± 1.0 | 0.35 ± 0.9 | -0.06 ± 1.0 | 0.27 ± 0.9 | -0.66 ± 1.0 | -0.26 ± 1.0 |
| LM-Ret | 0.09 ± 1.0 | 0.58 ± 0.7 | 0.09 ± 1.1 | 0.42 ± 0.9 | -0.49 ± 1.1 | 0.19 ± 0.9 |
| FAS | -0.09 ± 1.2 | 0.28 ± 1.4 | -0.02 ± 1.0 | -0.05 ± 1.0 | -0.55 ± 1.0 | -0.24 ± 1.1 |
| IED | -0.05 ± 1.0 | 0.18 ± 0.8 | -0.01 ± 1.1 | 0.19 ± 0.7 | -0.81 ± 1.5 | -0.66 ± 1.5 |
|  | PsySpd− (n = 36) | | SusAtn+ (n = 70) | | VerMem+ (n = 56) | |
|  | BL | FU | BL | FU | BL | FU |
| RVP-A | -0.13 ± 1.3 | -0.22 ± 1.2 | -0.75 ± 1.6 | 0.21 ± 1.2 | -0.46 ± 1.1 | -0.37 ± 1.2 |
| TMT-A | 0.53 ± 0.6 | -0.87 ± 1.6 | -0.16 ± 1.4 | 0.55 ± 0.8 | 0.04 ± 1.0 | 0.48 ± 0.8 |
| PAL | 0.26 ± 0.6 | -0.38 ± 1.1 | -0.23 ± 1.3 | 0.07 ± 1.0 | -0.60 ± 1.4 | -0.27 ± 1.2 |
| LM-I | 0.36 ± 1.1 | 0.18 ± 1.0 | -0.16 ± 1.0 | 0.38 ± 0.9 | -0.49 ± 0.9 | -0.02 ± 0.9 |
| LM-Ret | 0.44 ± 0.7 | 0.44 ± 0.9 | 0.30 ± 1.0 | 0.22 ± 0.9 | -0.76 ± 0.9 | 0.72 ± 0.6 |
| FAS | 0.13 ± 1.3 | 0.19 ± 1.5 | -0.16 ± 1.0 | -0.22 ± 1.1 | -0.35 ± 1.1 | 0.30 ± 1.1 |
| IED | 0.11 ± 1.0 | -0.05 ± 1.0 | -0.09 ± 1.0 | 0.07 ± 1.0 | -0.47 ± 1.4 | -0.02 ± 1.1 |

Abbreviations: FAS = Controlled Oral Word Association Test. IED = Intra-/Extradimensional Shift. LM-I = Logical Memory I. LM-Ret = Logical Memory II Percent Retention. PAL = Paired Associates Learning. RVP-A = Rapid Visual Processing. TMT-A = Trail Making Test – Part A.
