# Supplementary material for: Neuropsychological and functional outcomes in recent-onset major depression, bipolar disorder and schizophrenia-spectrum disorders: a longitudinal cohort study
Source: Transl Psychiatry. 2015 Apr 28;5(4):e555–. doi: 10.1038/tp.2015.50 (PMC4462613; doi:10.1038/tp.2015.50)
Supplement: Supplementary Table 3 [file tp201550x3.docx]

**SUPPLEMENTARY TABLE 3.** Diagnostic breakdown by clusters.

| Diagnosis x cluster ^a^ | PsySpd− | | SusAtn+ | | VerMem+ | |
| --- | --- | --- | --- | --- | --- | --- |
|  | n | % | n | % | n | % |
| MD | 18 | 26.1 | 26 | 37.7 | 25 | 36.2 |
| BD | 14 | 23.3 | 29 | 48.3 | 17 | 28.3 |
| SZ | 4 | 12.1 | 15 | 45.5 | 14 | 42.4 |

BD = Bipolar Disorder. MD = Major Depression. SZ = Schizophrenia-Spectrum Disorders.

^a^ *P* = 0.37.
