# Supplementary figures and images for: Neuropsychological and functional outcomes in recent-onset major depression, bipolar disorder and schizophrenia-spectrum disorders: a longitudinal cohort study
Source: Transl Psychiatry. 2015 Apr 28;5(4):e555–. doi: 10.1038/tp.2015.50 (PMC4462613; doi:10.1038/tp.2015.50)

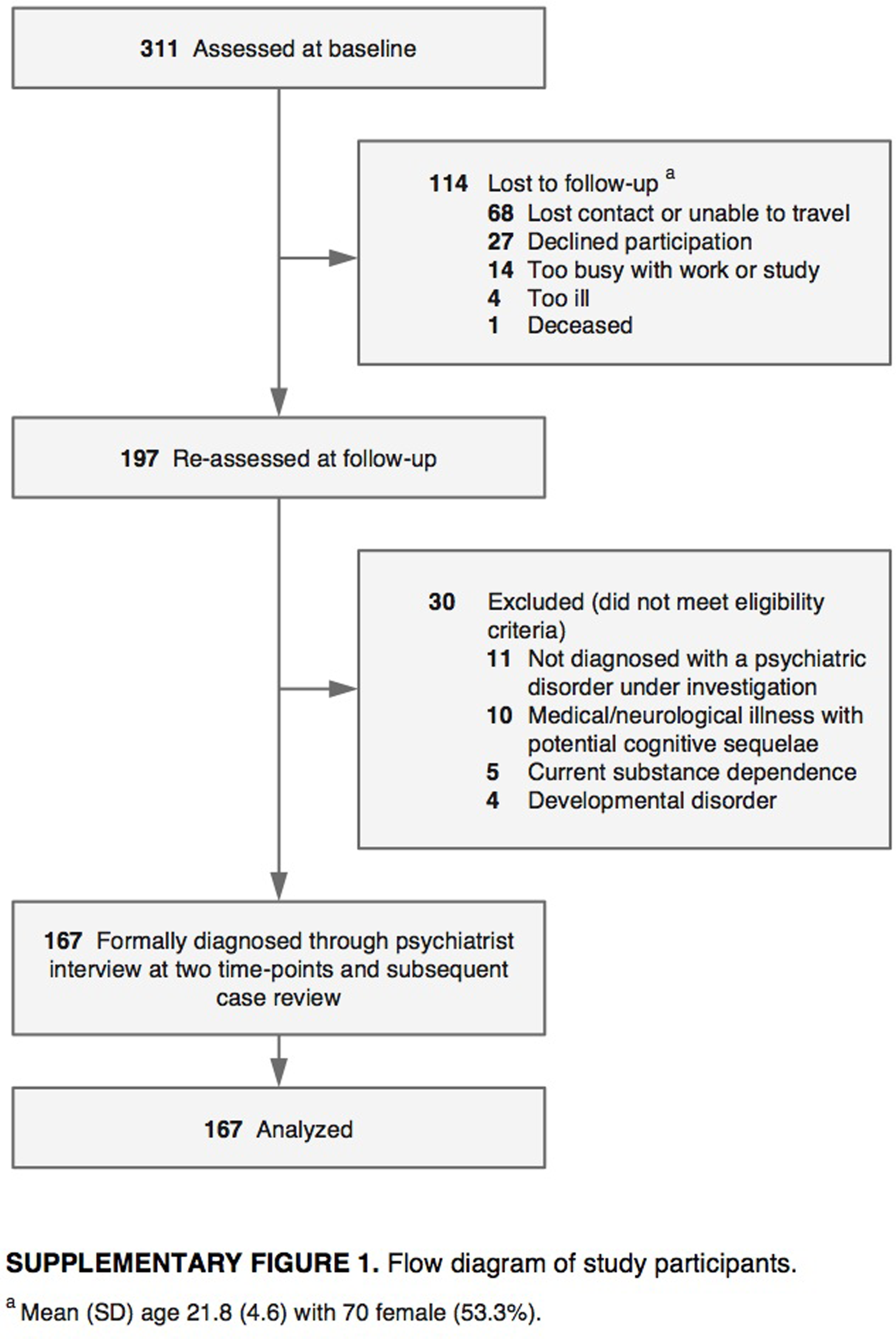

Supplement: Supplementary Figure 1 [file tp201550x4.tif]

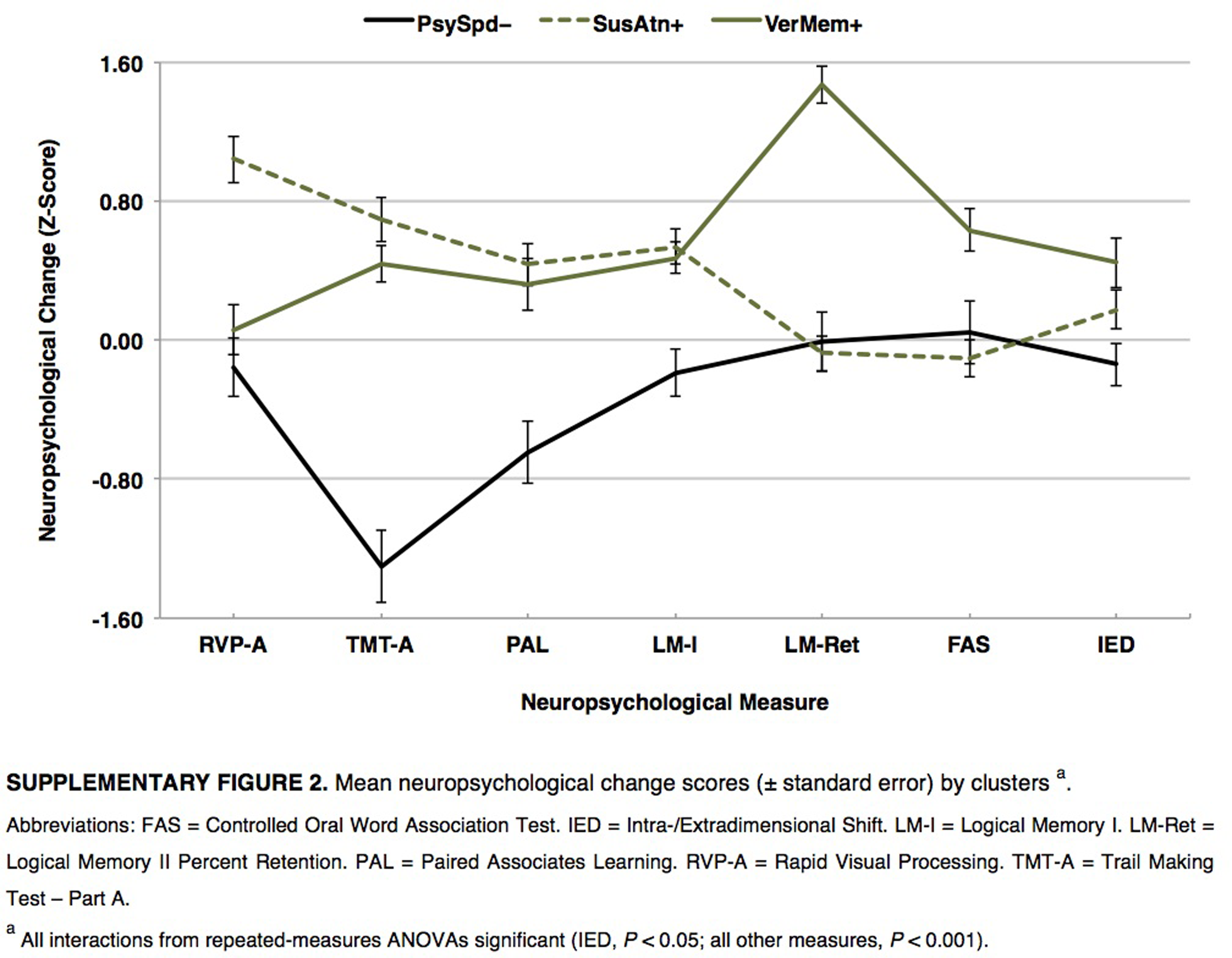

Supplement: Supplementary Figure 2 [file tp201550x5.tif]

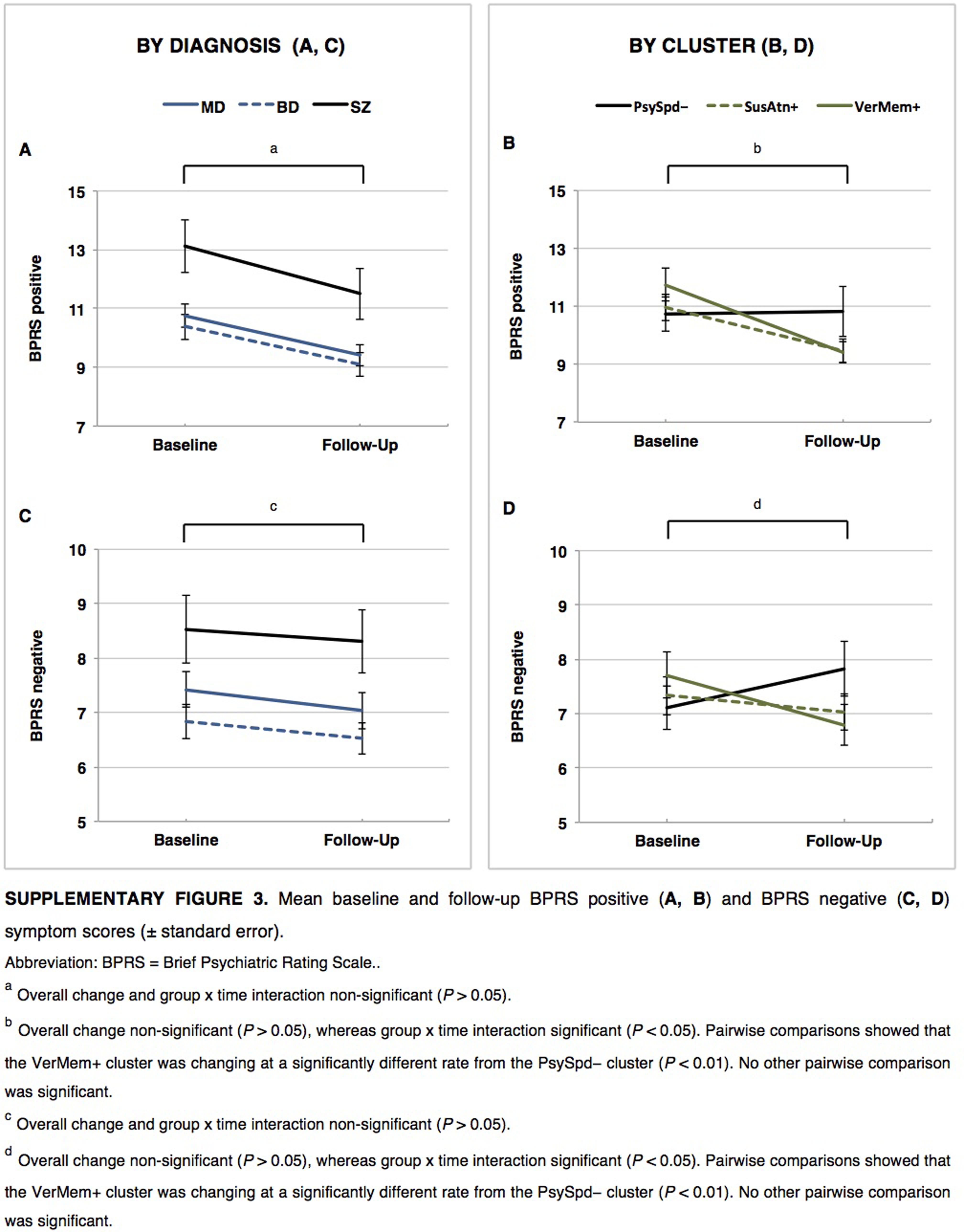

Supplement: Supplementary Figure 3 [file tp201550x6.tif]
